# Clinical and radiological features of pseudoprogression in brain tumors treated with immune checkpoint inhibitors

**DOI:** 10.1007/s11060-025-05091-0

**Published:** 2025-05-27

**Authors:** Maria José Ibáñez-Juliá, Luis Bataller, Francisco Javier Cabello-Murgui, Ludovic Nguyen-Them, Agusti Alentorn, Alba Torres-Martínez, Miguel Mazón-Momparler, Regina Gironés-Sarrió

**Affiliations:** 1 Neuroscience Department Ascires Biomedical Group, Valencia, Spain; 2 Department of Neurology, Hospital Universitari i Politècnic La Fe, Valencia, Spain; 3 Department of Neurology, Instituto Valenciano de Oncología, Valencia, Spain; 4 Sorbonne Universités, CNRS, UMR S 1127, Institut du Cerveau et de la Moelle épinière, Inserm, Paris, HP France; 5 Department of Neurology Mazarin, Hôpitaux universitaires Pitié-Salpêtrière Charles Foix.Assistance Publique Hôpitaux de Paris (APHP), Paris, France; 6 Medical Oncology Department, Hospital Universitari i Politècnic la Fe, Valencia, Spain; 7 Department of Radiology, Hospital Universitari i Politècnic La Fe, Valencia, Spain

**Keywords:** Pseudoprogression, Brain tumor, Checkpoint inhibitors, Immunotherapy

## Abstract

**Purpose:**

Immune checkpoint inhibitors (ICIs) are increasingly used in cancer treatment, resulting in the emergence of various immune-related adverse effects, including pseudoprogression (PsP). We sought to evaluate the characteristics of pseudoprogression in adults treated with ICIs for brain tumors (either primary or secondary), and to compare it with a non- PsP group.

**Methods:**

We retrospectively identified adults with brain tumors treated with ICIs at our institution between 2015 and 2023. Eligibility required one brain magnetic resonance imaging scan prior to treatment and another obtained within 6 months after treatment initiation. PsP was defined as radiological worsening within 6 months of ICI initiation, followed by stabilization or improvement without therapy modification. Demographic, clinical, and radiological characteristics were analyzed and compared between the PsP and the non-PsP groups.

**Results:**

Among 102 eligible patients, 10 (9.8%) developed PsP. Clinical symptoms occurred in 4 (40%) cases, all of which showed favorable outcomes with corticosteroid therapy. The PsP group had higher baseline tumor burden (*p* = 1.29 × 10⁻¹³) and higher PD-L1 expression (*p* < 0.001) than the non-PsP group. Median progression-free survival and overall survival were numerically longer in the PsP group with no significant difference.

**Conclusions:**

PsP is a frequent complication of ICIs. We describe 4 symptomatic patients with pseudoprogression, challenging the iRANO criteria that recommend excluding this diagnosis in symptomatic cases. Clinical impairment should not automatically rule out pseudoprogression, and each case requires thorough evaluation. High PD-L1 expression and greater tumor burden may be associated with PsP, but further studies are needed to confirm these findings.

**Supplementary Information:**

The online version contains supplementary material available at 10.1007/s11060-025-05091-0.

## Introduction

Cancer cells can evade immunosurveillance by triggering inhibitory pathways of T cell activation. Key components of these inhibitory checkpoints include cytotoxic T-lymphocyte-associated antigen 4 (CTLA-4), programmed cell death protein 1 (PD-1), and its ligand PD-L1. Immune checkpoint inhibitors (ICIs) block these inhibitory pathways, thus unleashing antitumor immune response [1].

In the past decade, ICIs have revolutionized the treatment of systemic tumors, such as melanoma and lung cancer [2–4] and, as they can cross the blood-brain barrier (BBB) they are also an effective treatment for brain metastases [5].

In brain tumors, pseudoprogression (PsP) is one of the complications associated with ICIs, characterized by initial radiological impairment caused by inflammatory infiltrates rather than an increase in tumor cell infiltration. Follow-up images typically show a decrease or stability in tumor burden [6].

In clinical practice, PsP can be misinterpreted as real progression, potentially leading to unnecessary changes in cancer therapy. Therefore, correct recognition of this complication is essential to provide the most suitable treatment.

Due to the variable and often unpredictable radiological response to immunotherapy, the Response Assessment in Neuro-Oncology (RANO) criteria were no longer adequate for evaluating brain tumor response in those patients. Consequently, the Immunotherapy Response Assessment in Neuro-Oncology (iRANO) criteria were developed to assess a more accurate evaluation of immunotherapy responses in brain tumors. Briefly, the iRANO criteria define PsP as radiographic worsening within the first 6 months of immunotherapy, without clinical deterioration, that stabilizes or improves without treatment changes, as confirmed by follow-up imaging [7]. Nevertheless, there are still many unanswered questions regarding the assessment and characteristics of PsP. To date, cerebral PsP has been reported in only one large cohort and a few case reports or small series involving brain metastases treated with immunotherapy [6, 8–10], whereas studies in diffuse gliomas mainly describe PsP after chemoradiotherapy rather than ICI treatment [11].

To our knowledge, no studies have been published comparing a group of patients with brain PsP to a non-PsP group.

In this paper, we sought to evaluate the incidence and characteristics of PsP in adults treated with ICIs for brain tumors (primary or secondary), and to compare it to a non-PsP group. We performed a single-institution observational study of all patients with brain tumors and available cerebral MRI treated with ICIs.

## Materials and methods

We performed a retrospective analysis of patients treated in our hospital between 2015 and 2023 with any ICI treatment, including PD-1 inhibitors (Pembrolizumab, Nivolumab, Cemiplimab), PD-L1 inhibitors (Atezolilumab, Durvalumab, Avelumab) and CTLA-4 inhibitors (Ipilimumab).

We selected patients meeting the following inclusion criteria:

1. Diagnosis of brain tumor (primary or metastatic), regardless of tumor histology.
2. Treated with ICIs.
3. Age ≥ 18 years at the time of ICIs administration.
4. Available brain MRI both before treatment and within six months after starting ICIs.

Brain MRIs were performed on 1.5 or 3 T scanners and included T1-weighted sequences with and without contrast, and T2-weighted sequences with fluid-attenuated inversion recovery (FLAIR). All MRIs were analyzed by expert neuroradiologists.

Tumor burden was assessed on pre- and post-treatment MRI scans. It was quantified by manually delineating the enhancing area on the axial slice with the largest cross-sectional diameter, in contrast-enhanced T1-weighted (CET1) sequences. Area was calculated in mm². Measurable disease was defined according to iRANO criteria as enhancing lesions ≥ 10 mm in maximum diameter [7].

According to the iRANO criteria, PsP was defined as radiological worsening within six months of ICI initiation, with stability or improvement in subsequent follow-up, without any treatment modifications [7]. Radiological worsening and improvement were defined as new or increased contrast-enhancing lesions on CET1 MRI sequences and/or increased edema on T2/FLAIR sequences, and their subsequent stabilization or reduction, respectively. The iRANO criterion of significant clinical decline was not considered a formal exclusion criterion. In those particular cases, the diagnosis of pseudoprogression was primarily based on radiological features (mainly increased perilesional edema or the appearance of new distant lesions), the timing of symptom onset relative to the initiation of ICI therapy, and the overall response of the systemic disease.

We considered ICI to be combined with radiotherapy when the interval between the first dose of radiation and the first infusion was less than one month.

Total radiation dose was calculated by summing the prescribed doses from all RT modalities received by each patient. Due to the retrospective design and heterogeneity of techniques, a more precise estimation was not feasible.

Corticosteroid doses at ICI initiation were recorded, and patients stratified by dose (< 2 mg vs. ≥2 mg dexamethasone or equivalent), given the potential impact of higher doses on ICI efficacy.

PD-L1 expression on tumor cells was assessed using the PD-L1 IHC 22C3 pharmDx assay and reported as tumor proportion score (TPS).

Clinical reports of all included patients were retrospectively reviewed, and clinical and radiological data were collected. For patients diagnosed with PsP, particular attention was paid to MRI patterns and clinical outcomes.

### Statistical analysis

Data were summarized by median and interquartile ranges (IQR) for continuous variables and by frequency and percentage for categorical variables. Patients characteristics were compared using the Wilcoxon or Student-t test for continuous variables and the Chi square test or Fisher’s exact test for categorical variables, depending on sample size and data distribution. Survival analyses were performed using the Kaplan-Meier method and compared using the log-rank test. A *p*-value of ≤ 0.05 was considered statistically significant.Analyses were performed using RStudio (RStudio, PBC, Boston, MA).

#### Ethics approval

Ethics approval for this retrospective data collection was obtained from the ethics committee of the University Hospital La Fe, in Valencia, Spain.

## Results

**Fig. 1:**
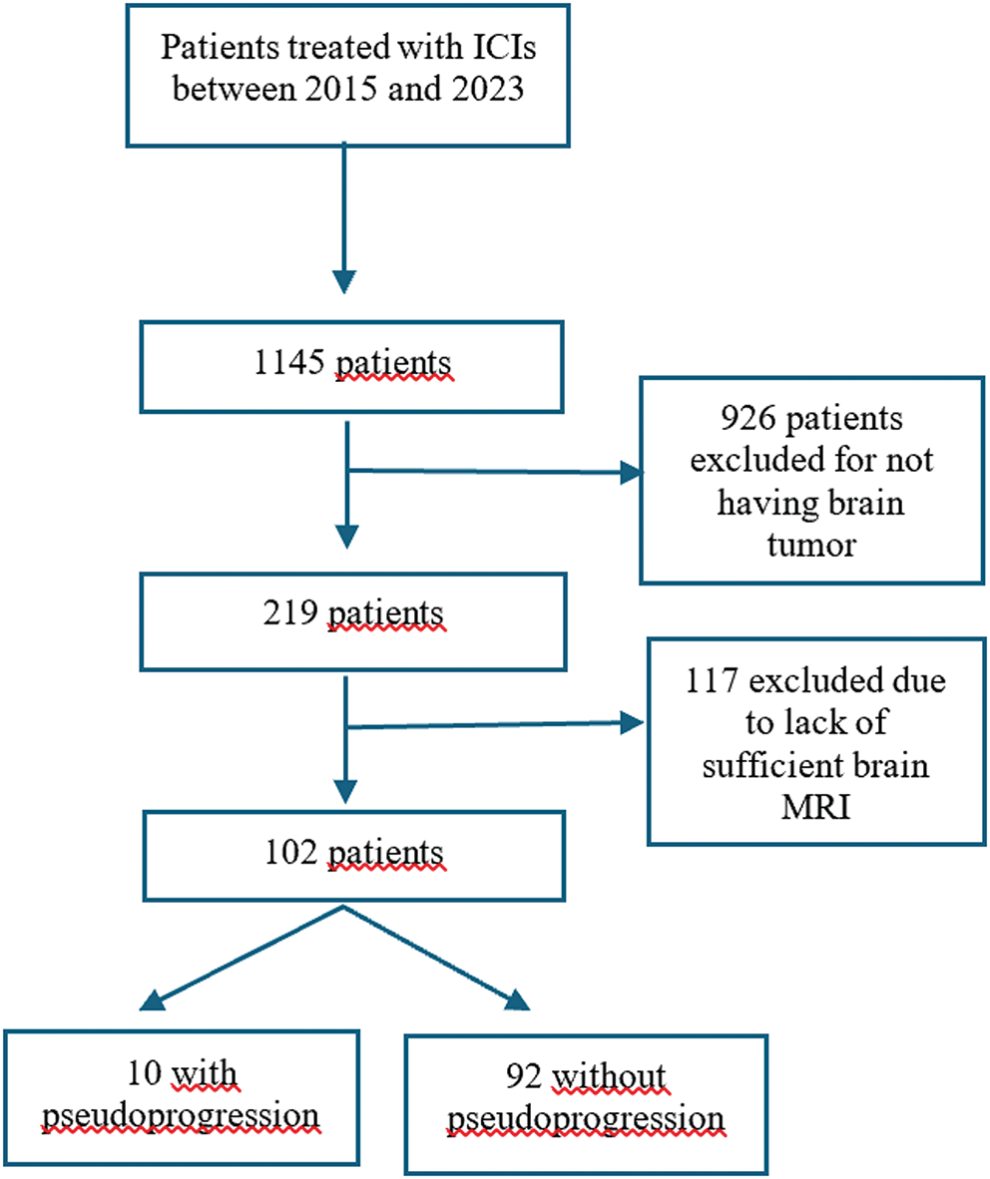
Consort flowchart

A total of 1145 patients were treated with ICIs between 2015 and 2023, and 102 of them met the inclusion criteria. The flowchart is represented in Fig. 1.

### Patients with pseudoprogression

Among the 102 patients, 10 were considered to have PsP, representing a PsP rate of 9.8%.

The main characteristics of the patients with PsP are summarized in Table 1.

**Table 1:** Characteristics of patients with pseudoprogression

	Sex/Age	Cancer	ICI	% PDL1 expression	RT Type	Combined RT-ICI	Symptoms	MRI Pattern	Delay of PsP	Management
1	M / 52	Lung adenocarcinoma	Pembrolizumab	90	WBRT+FRT	No	No	Increased contrast enhancement and edema	16 days	Dexamethasone 4 mg/day, already initiated before PsP; no dose adjustment.
2	M / 46	Lung adenocarcinoma	Pembrolizumab	90	SBRT	Yes	Somnolence Headache	Increased contrast enhancement and edema	6 days	Dexamethasone 12 mg bolus rapidly tapered to 4 mg/day orally
3	M / 54	Lung adenocarcinoma	Pembrolizumab	70	SBRT + SRS	Yes	No	New lesions	53 days	None
4	M / 75	Lung adenocarcinoma	Pembrolizumab	50	SBRT	Yes	No	New lesions	64 days	None
5	F / 60	Lung adenocarcinoma	Nivolumab	NA	SBRT + SRS	No	No	Increased contrast enhancement and edema	101 days	Dexamethasone 6 mg/day with 6-day tapering protocol
6	M / 48	Melanoma	Nivolumab/Ipilimumab	NA	SBRT	No	No	Increased contrast enhancement and edema	63 days	Dexamethasone 4 mg IV for 3 days, rapidly tapered to 2 mg/day orally
7	M / 64	Melanoma	Nivolumab	NA	SBRT	Yes	No	New lesions	37 days	Dexamethasone 2 mg/day already initiated before PsP; no dose adjustment.
8	F / 55	Lung adenocarcinoma	Nivolumab/Ipilimumab	5	SRS	No	Dysarthria, right hemiparesis	Increased contrast enhancement and edema	11 days	Dexamethasone 4 mg every 12 h, rapidly tapered to 2 mg/day
9	M / 47	Lung adenocarcinoma	Nivolumab/Ipilimumab	0	SBRT	No	Aphasia, right hemiparesis	Increased contrast enhancement and edema	15 days	Dexamethasone 4 mg every 12 h, rapidly tapered to 2 mg/day
10	M / 53	Anaplastic Gangliocytoma	Nivolumab/Ipilimumab	NA	FRT	No	Aphasia, right hemiparesis	Increased contrast enhancement and edema	31 days	Dexamethasone 8 mg/day rapidly tapered to 2 mg/day

F = Female; FRT = Fractionated radiotherapy; ICI = immune checkpoint inhibitors; M = Male; NA = Non available; PsP = Pseudoprogression; RT = Radiotherapy; SBRT = Stereotactic brain radiotherapy; SRS = Stereotactic Radiosurgery; WBRT = Whole Brain Radiotherapy

Within this group of 10 patients, there were 8 males and 2 females, with a median age at diagnosis of 53.5 years (IQR: 49-58.75). Seven (70%) patients had lung adenocarcinoma, 2 (20%) melanoma, and 1 (10%) anaplastic gangliocytoma.

Regarding treatment, 4 patients (40%) received pembrolizumab, 2 (20%) nivolumab, and 4 (40%) a combination of nivolumab and ipilimumab.

PD-L1 expression was greater than 50% in 4 patients (40%), negative in 2 (20%), and not available in 4 (40%).

All patients received radiotherapy, which was administered in combination with ICI in 4 cases (40%). Stereotactic brain radiotherapy (SBRT) was the most commonly used RT modality (7 patients, 70%), administered alone (Patients 2, 5, 6, 7, and 9) or in combination with stereotactic radiosurgery (SRS) (Patients 3 and 4). Patient 1 received whole-brain radiotherapy (WBRT) combined with fractionated RT (FRT). Patient 8 underwent SRS for a single lesion, and patient 10 received FRT for a gangliocytoma. PsP occurred within the irradiated field in 7 cases, whereas in 3 patients, new lesions emerged outside the irradiated area.

PsP was symptomatic in 4 (40%) cases due to mass effect. Median time from ICI initiation to PsP occurrence was 34 days, and it appeared sooner in the symptomatic subgroup compared to the asymptomatic group (13 vs. 58 days, *p* = 0.002). Interestingly, 3 out of the 4 patients treated with a combination of nivolumab ipilimumab were symptomatic.

Median PFS and OS were longer in asymptomatic patients compared to the symptomatic group (median PFS: 13.9 vs. 3.9 months, respectively; median OS, 26.5 vs. 11.4 months, respectively). However, no statistically significant differences were observed for either PFS (hazard ratio (HR), 2.65; 95% confidence interval (CI), 0.42–16.68; *p =* 0.3) or OS (HR, 4.18; 95% CI, 0.67–26.24; *p* = 0.1).

Two radiological patterns of PsP were found:

1. In patients 1, 2, 5, 6, 8, 9 and 10, the follow-up MRI showed increased contrast enhancement in pre-existing lesions on CET1 sequences, and in the surrounding edema on T2-weighted images.
2. In Patients 3, 4, and 7, follow-up MRI revealed multiple new contrast-enhancing lesions on CET1 sequences, each < 1 cm in diameter with mild edema, located in different territories and distant from preexisting lesions, some outside the radiation field.

**Fig. 2:**
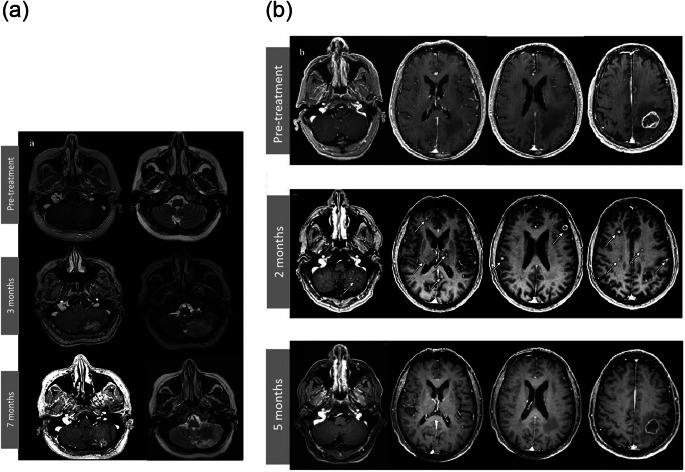
**a**: Post-contrast T1- and T2-weighted images in a patient with a left cerebellar metastatic lesion taken before treatment initiation, after 3 months of ICI treatment, and after 7 months of ICI treatment. Brain MRI after 3 months of treatment showed increased contrast enhancement and edema, which spontaneously improved 4 months later. **b**: Post-contrast T1 weighted images in a patient with an initial left parietal metastatic lesion taken before treatment initiation, after 2 months of ICI treatment, and after 5 months of ICI treatment. Brain MRI after 2 months of treatment showed multiple new disseminated lesions (white arrows), which spontaneously disappeared 3 months later

Figures 2a and b illustrate the 2 most representative patterns of PsP.

A description of tumor burden evolution in the 10 patients with PsP is provided in supplementary material 1.

### Pseudoprogression VS Non pseudoprogression group

Demographic and clinical characteristics of all the 102 patients are summarized in Table 2.

**Table 2:** Baseline characteristics in patients

Variable	PsP group (*n* = 10)	No PsP group (*n* = 92)	*p*-value (PsP vs. non-PsP)
**Median age at diagnosis**,** years (IQR)**	53.5 (49-58.75)	59 (53–65)	0.2
**Gender**,** N (%)**			0.33
Male	8 (80%)	60 (65%)	
Female	2 (20%)	32 (35%)	
**Type of tumor**,** N (%)**			0.98
Primary CNS tumor	1 (10%)	9 (10%)	
Metastatic tumor	9 (90%)	83 (90%)	
**Tumor histology**,** N (%)**			0.24
Lung adenocarcinoma	7 (70%)	52 (56%)	
Small cell lung cancer	0 (0%)	8 (9%)	
Other NSCLC	0 (0%)	9 (10%)	
Melanoma	2 (20%)	7 (8%)	
Clear Cell Renal Cell Carcinoma	0 (0%)	4 (4%)	
Other	1 (10%)	12 (13%)	
**Median pre-treatment tumor burden**,** mm2 (IQR)**	424 (116-1029.5)	169 (0-408)	1.29 × 10^− 13^
**PD-L1 expression**			0.0005
≥ 50%	4 (40%)	17 (18%)	
1–49%	1 (10%)	16 (17%)	
Negative	1 (10%)	29 (32%)	
NA	4 (40%)	30 (33%)	
**ICI treatment**,** N (%)**			0.38
Nivolumab	2 (20%)	15 (16%)	
Pembrolizumab	4 (40%)	34 (37%)	
Atezolizumab	0 (0%)	21 (23%)	
Ipilimumab	0 (0%)	1 (1%)	
Nivolumab/Ipilimumab	4 (40%)	16 (17%)	
Other	0 (0%)	5 (6%)	
**Prior systemic treatment**,** N (%)**			0.79
ICIs	1 (10%)	2 (2%)	
TT	1 (10%)	10 (11%)	
Chemotherapy	4 (40%)	37 (40%)	
**RT**,** N (%)**			0.49
Prior to ICI	6 (60%)	42 (46%)	
Concomitant	4 (40%)	37 (40%)	
No RT	0 (0%)	13 (14%)	
**Median total dose of RT**,** Gy (IQR)**	36.25 (30–63)	30 (27.75–56.25)	2.39 × 10⁻⁸
**RT modality**,** N (%)**			0.49
Radiosurgery	2 (20%)	16 (17%)	
SBRT	7 (70%)	36 (39%)	
WBRT	1 (10%)	25 (26%)	
Fractioned RT	2 (20%)	17 (18%)	
**Concomitant Corticotherapy**,** N (%)**	5 (50%)	29 (32%)	0.44
Dexamethasone <2 mg/day, N (%)	0 (0%)	12 (41%)	
Dexamethasone ≥ 2 mg/day N (%)	5 (100%)	17 (59%)	0.14

CNS = Central nervous system; IQR = Interquartile range; NSCLC = Non small cell Lung Cancer; PsP = Pseudoprogression; RT = Radiotherapy; SBRT = stereotaxic brain radiotherapy; TT = Targeted Therapy; WBRT = whole brain radiotherapy. P-values for multi-category variables correspond to overall group comparisons

Median age at cancer diagnosis was 53.5 (IQR: 49–58,75) in the PsP group and 59 years (IQR: 53–65) in the non-PsP group, with a male predominance in both groups (80% and 65% respectively). Primary central nervous system (CNS) tumors represented 10% of cases, and metastases 90% in both groups. Lung cancer, especially adenocarcinoma, was the most frequent primary tumor (70% in PsP VS. 75% in non-PsP).

Of the 102 patients, 67 had measurable disease per iRANO criteria at baseline (8 in the PsP group, 59 in the non-PsP group). Median tumor burden on pre-treatment brain MRI was higher in the PsP group compared to the non-PsP group (424 mm², IQR 116–1029.5 vs. 169 mm², IQR 0–408, respectively; *p* = 1.29 × 10⁻¹³).

PDL1 expression was higher in the PsP group with a median PDL1 expression of 70% in the PsP group vs. 1,5% in the non PsP group (*p* < 0.001).

Combined radiotherapy and ICI use did not differ significantly between groups (*p* = 0.49), and analysis of the different radiotherapy modalities also revealed no significant differences (*p* = 0.49). Nevertheless, the median total RT dose was higher in the PsP group (36.25 Gy, IQR: 30–63) than in the non-PsP group (30 Gy, IQR:27.75–56.25) (*p* = 2.391 × 10⁻⁸).

There were no significant differences between groups regarding corticosteroid use at ICI initiation (50% in the PsP group vs. 32% in the non-PsP group; *p* = 0.44). Similarly, no significant differences were observed when patients were stratified by corticosteroid dose (*p* = 0.14).

Concerning ICI treatment, out of 20 patients treated with the combination of nivolumab and ipilimumab, 4 (20%) experienced PsP, which represents a higher rate than that of pembrolizumab (4/38, 11.7%) or nivolumab alone (2/17, 10.5%). Nevertheless, when comparing the proportion of patients receiving the ipilimumab/nivolumab combination between the PsP and non-PsP groups (40% vs. 17%, respectively), this difference was not statistically significant (*p* = 0.1).

**Fig. 3:**
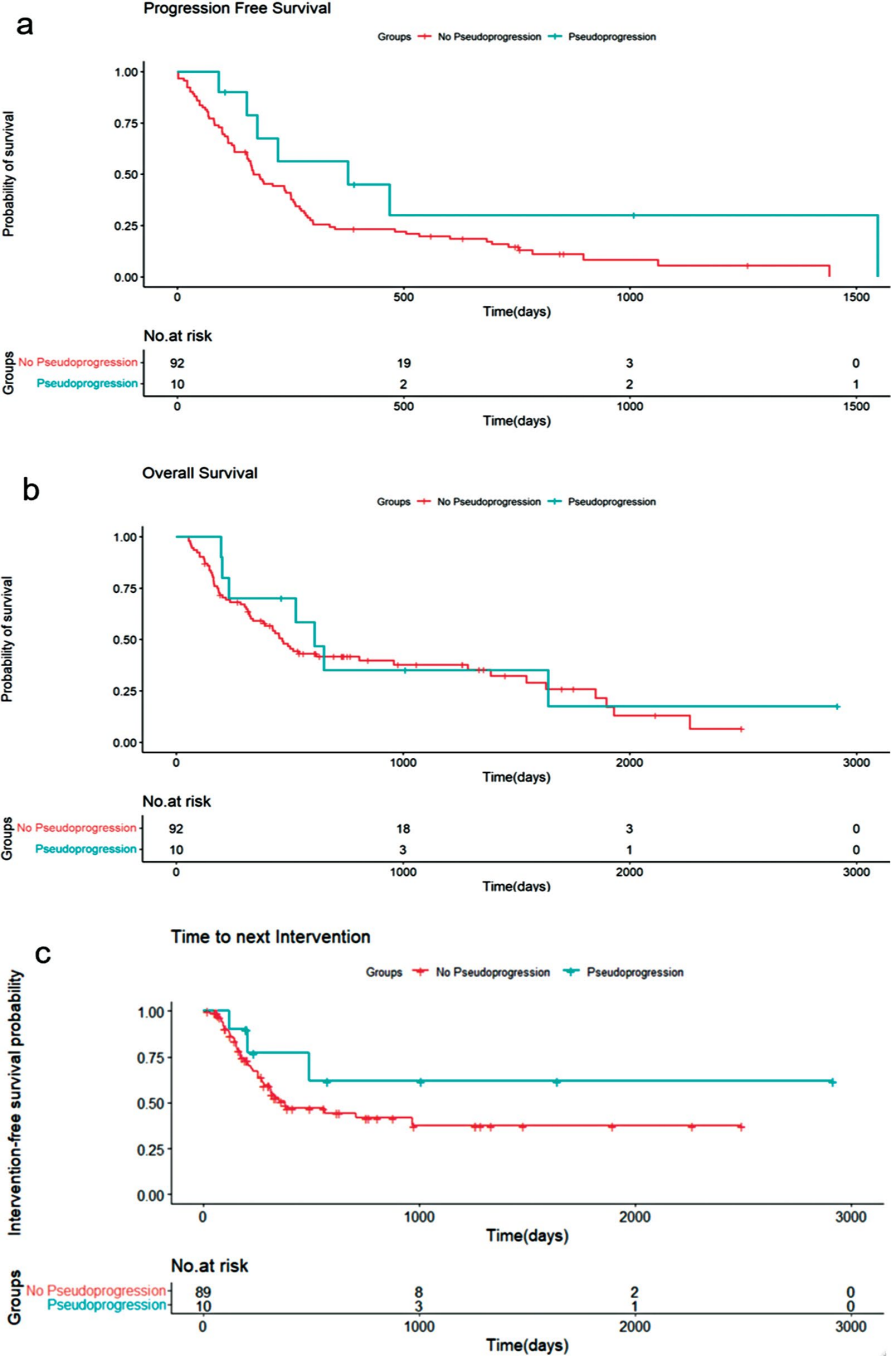
Progression free survival (**a**), Overal survivall (**b**), and Time to next intervention (**c**) in patients with and without PsP

Progression-free survival (PFS), overall survival (OS), and time to next intervention (TTNI) were all numerically longer in the PsP group compared to the non-PsP group, although none of the differences reached statistical significance. Median PFS was 9.8 vs. 5.5 months (HR, 0.47; 95% CI, 0.20–1.07; *p* = 0.05), median OS was 18.6 vs. 9.9 months (HR, 0.78; 95% CI, 0.36–1.7; *p* = 0.5), and median TTNI was 11.9 vs. 8.3 months (HR, 0.50; 95% CI, 0.16–1.63; *p* = 0.25) (Figs. 3a–c). Furthemore, longer (TTNI) was significantly associated with improved OS (HR, 0.99; 95% CI, 0.9969–0.9988; *p* = 1.46 × 10⁻⁵), suggesting that patients who required a new intervention later had more favorable outcomes, likely reflecting better initial treatment response.

A supplementary analysis in the lung cancer subgroup yielded findings similar to those of the overall cohort. Detailed results are available in the Supplementary Material 2.

## Discussion

Immune checkpoint inhibitors are widely used for the treatment of systemic cancers, particularly lung cancer and melanoma [2–4]. As they can cross the BBB, ICIs are also effective in the treatment of brain metastases, and they have also been used for the treatment of some primary brain tumors [5, 12]. While PsP has been extensively studied in diffuse gliomas treated with chemoradiotherapy, evidence in brain tumors treated with ICIs remains limited, consisting mostly of case reports and small series [6, 8–10].

The iRANO criteria define PsP as radiological worsening within the first six months of ICI therapy, without clinical impairment, followed by stability or improvement on subsequent MRI. Consequently, any clinical worsening accompanied by radiological progression should be interpreted as true progression [7]. In our study, we considered that any brain mass effect could cause neurological symptoms; thus, clinical worsening was not considered a formal exclusion criterion for PsP, and in those particular cases, the diagnosis of PsP versus true progression was individualized based on both radiological and clinical features.

Four of the 10 PsP patients experienced symptoms related to brain mass effect. They were treated with corticosteroids, and ICI therapy was continued. Follow-up brain MRI eventually demonstrated radiological stability or improvement, accompanied by clinical improvement. Furthemore, no differences were observed between symptomatic and asymptomatic patients regarding PFS and OS. These findings support the idea that clinical impairment should not be systematically considered an exclusion criterion for PsP. When present, each case should be carefully evaluated by a multidisciplinary expert committee.

Intriguingly, in our series, we observed a higher proportion of pseudoprogression in patients treated with the combination of nivolumab and ipilimumab compared to those receiving monotherapy. Although there is no statistically significant difference (*p* = 0.1), this finding suggests that PsP may be more common with combination ICI therapy than with ICI monotherapy, as has been suggested in previous clinical reports [9].

Some studies report a higher incidence of immune-related adverse effects (irAEs) with anti–CTLA-4 agents compared to PD-1/PD-L1 antibodies [13, 14] and the incidence and severity of irAEs are further increased when ICIs are used in combination [15]. Although PsP is not specifically mentioned, since it is related to immune system upregulation, it is logical to assume that it may be more pronounced in the context of combination ICI therapy. Thus, if these results are confirmed, special attention should be given to patients receiving ICI combinations, considering the higher risk of PsP in cases of radiological changes.

In univariate analysis, PD-L1 expression was higher in the PsP group (*p* < 0.001). PD-L1 protein expression is the main predictive biomarker for immunotherapy response, particularly in lung cancer. Tumor cells express PD-L1 protein as a mechanism to evade the immune system’s antitumor response. Consequently, tumors with higher levels of PD-L1 expression may trigger a stronger immune response upon inhibition of the PD-1 pathway. High expression levels are therefore associated with a better response to immunotherapy [16, 17]. Sugisaka et al. reported that high PD-L1 expression (≥ 50%) was a predictive factor of irAE too [18]. Similarly, high PD-L1 expression could be a biomarker for PsP, as it promotes immune response and leads to subsequent inflammatory infiltration.

Tumor burden was higher in the PsP group (*p* = 1.29 × 10⁻¹³). A review by Morrissey et al. found no association between baseline tumor volume and PsP risk in systemic tumors treated with immunotherapy [19]. Similarly, in primary CNS tumors treated with chemoradiotherapy, no statistically significant differences in baseline tumor volume between patients with true progression and those with pseudoprogression have been reported [20, 21]. To our knowledge, this is the first study to assess baseline tumor volume as a possible risk factor for pseudoprogression in CNS tumors treated with ICIs. These findings should be interpreted with caution, and further studies incorporating lesion-specific volumetric analysis are needed to confirm our observations.

Patients with brain PsP demonstrated numerically longer PFS, OS and TTNI than those without PsP, without significant difference found. Previous studies in systemic tumors reported better outcomes in patients with PsP [22, 23]. Moreover, PsP is considered as a good prognostic factor in primary CNS tumors treated with chemoradiotherapy [24].

This is a key point, as the occurrence of PsP in the early phase of therapy may lead to premature discontinuation of ICIs, due to a perceived lack of efficacy, or unnecessary brain irradiation. Oncologists should be aware that the therapeutic effect may occur later than the onset of PsP, and current evidence supports a positive association between irAEs and survival outcomes [25–27].

### Limitations

This retrospective study has several limitations that should be acknowledged. The main limitation is the small sample size of the PsP group, requiring cautious interpretation of the results. In order to better understand PsP behavior and identify potential predisposing factors, further studies with larger sample sizes must be conducted in the future. In addition, all brain tumors, regardless of the primary systemic tumor type, were included, leading to significant heterogeneity in the samples, limiting definitive conclusions.

It is important to note that pseudopogression is a well known complication of radiotherapy and, in our series, all patients with PsP received RT prior to ICI administration, which make difficult to confirm the role of ICI in PsP. Nevertheless, 3 out of the 10 patients presented with new lesions, distant from the radiation field. For the remaining 7 patients, a contribution of RT to the development of PsP could not be excluded. Nevertheless, given that RT is a cornerstone in the treatment of CNS tumors, it is challenging to isolate the role of ICI alone in the development of PsP in these cases.

Even though no formal conclusions can be drawn due to the small sample size and its heterogeneity, our results may lay the groundwork for the design of future studies.

## Conclusions

To our knowledge, this is the first study on brain tumor PsP in patients treated with ICIs comparing a PsP group with a non-PsP group. We report 4 symptomatic cases of PsP treated with corticosteroids, showing clinical improvement and stable or improved radiological findings in successive follow-up brain MRIs. Contrary to iRANO recommendations, in our opinion, clinical impairment should not systematically exclude PsP, and each case should be carefully evaluated.

Although our study has several methodological limitations, we observed that increased PD-L1 expression, higher baseline tumor burden, and combined ICI use were more frequent in the PsP group.

These findings, while preliminary, may help raise clinical suspicion of PsP and support individualized management strategies to avoid unnecessary treatment modifications.

## Electronic supplementary material

Below is the link to the electronic supplementary material.

Supplementary Material 1

Supplementary Material 2

## Data Availability

Data is provided within the manuscript or supplementary information files.
