# Supplementary material for: Clinical and radiological features of pseudoprogression in brain tumors treated with immune checkpoint inhibitors
Source: J Neurooncol. 2025 May 27;174(3):779–88. doi: 10.1007/s11060-025-05091-0 (PMC12263762; doi:10.1007/s11060-025-05091-0)
Supplement: Supplementary file 1 — Supplementary Material 1 [file 11060_2025_5091_MOESM1_ESM.docx]

Supplementary material 1.

Tumor burden evolution in 10 patients with PsP

Eight of 10 patients experienced an increase in tumor burden in the first MRI performed after ICI initiation. Among these eight patients, tumor burden subsequently decreased in five cases (Patients 1, 2, 3, 5, and 8), while it remained stable in the other three (Patients 4, 7, and 9). In Patient 4, tumor burden decreased despite PsP, likely due to a favorable response of the primary lesion, along with the appearance of new distant non-measurable lesions. In Patient 10, no measurable lesions were present.


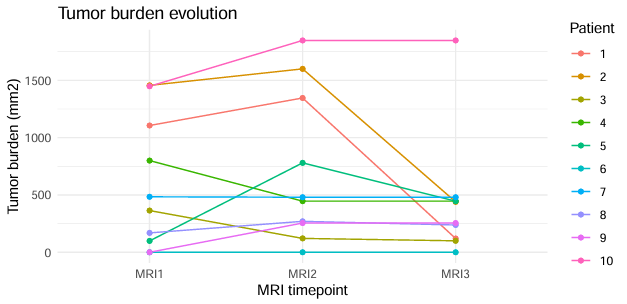


Supplementary Fig. 3 Tumor burden evolution in the 10 patients with PsP
