# Supplementary material for: Clinical and radiological features of pseudoprogression in brain tumors treated with immune checkpoint inhibitors
Source: J Neurooncol. 2025 May 27;174(3):779–88. doi: 10.1007/s11060-025-05091-0 (PMC12263762; doi:10.1007/s11060-025-05091-0)
Supplement: Supplementary file 2 — Supplementary Material 2 [file 11060_2025_5091_MOESM2_ESM.docx]

Supplementary material 2

Analysis of the lung cancer subgroup.

The lung cancer subgroup included 76 patients: 7 with PsP and 69 without PsP. Demographic and clinical characteristics of all 76 patients are summarized in Supplementary Table 1.

| **Variable** | **Pseudoprogression group (N=7)** | **No Pseudoprogression group (N= 69)** | ***p*-value (PsP vs. non-PsP)** |
| --- | --- | --- | --- |
| **Median age at diagnosis,**  **years (IQR)** | 54 (49,5-57.5) | 61 (55-65) | 1.663x10^-07^ |
| **Gender, N (%)** |  |  | 0.64 |
| Male | 5 (71%) | 44 (64%) |  |
| Female | 2 (29%) | 25 (36%) |  |
| **Tumor histology, N (%)** |  |  | 0.02 |
| Lung adenocarcinoma | 7 (100%) | 52 (75%) |  |
| Small cell lung cancer | 0 (0%) | 8 (12%) |  |
| Other NSCLC | 0 (0%) | 9 (13%) |  |
| **Median pre-treatment tumor burden, mm2 (IQR)** | 364 (134-953) | 110 (0-330) | 4.10×10^−10^ |
| **PD-L1 expression** |  |  | 0.00002 |
| ≥ 50% | 4 (58%) | 17 (24%) |  |
| 1-49% | 1 (14%) | 15 (22%) |  |
| Negative | 1 (14%) | 29 (42%) |  |
| NA | 1 (14%) | 8 (12%) |  |
| **ICI treatment, N (%)** |  |  | 0.34 |
| Nivolumab | 1 (14%) | 8 (12%) |  |
| Pembrolizumab | 4 (58%) | 32 (46%) |  |
| Atezolizumab | 0 (0%) | 16 (23%) |  |
| Nivolumab/Ipilimumab | 2 (28%) | 8 (12%) |  |
| Other | 0 (0%) | 5 (7%) |  |
| **Prior systemic treatment, N (%)** |  |  | 0.48 |
| ICIs | 0 (0%) | 1 (1%) |  |
| TT | 0 (0%) | 2 (2%) |  |
| Chemotherapy | 4 (58%) | 27 (39%) |  |
| **Median total dose of RT, Gy ( IQR)** | 40 (28-65) | 30 (27-53.25) | 3.3 × 10⁻7 |
| **RT, N (%)** |  |  | 0.55 |
| Prior to ICI | 4 (58%) | 27 (39%) |  |
| Concomitant | 3 (42%) | 32 (46%) |  |
| No RT | 0 (0%) | 10 (15%) |  |
| **RT modality, N (%)** |  |  | 0.48 |
| Radiosurgery | 3 (42%) | 9 (13%) |  |
| SBRT | 5 (70%) | 26 (37%) |  |
| WBRT | 1 (14%) | 23 (33%) |  |
| Fractioned RT | 1 (14%) | 17 (25%) |  |
| **Concomitant Corticotherapy, N (%)** | 5 (71%) | 19 (28%) | 0.03 (0.89-68.4) |

**Supplementary Table 1:** Baseline characteristics in patients.

CNS = Central nervous system; IQR = Interquartile ranges; NSCLC = Non small cell Lung Cancer; RT = Radiotherapy; SBRT = stereotaxic brain radiotherapy; TT = Targeted Therapy; WBRT = whole brain radiotherapy

P-values for multi-category variables correspond to overall group comparisons

Patients were younger in the PsP group compared to the non-PsP group (median age: 54 years, IQR: 49,5-57.5, vs. 61 years, IQR: 55–65; *p* = 1.66 × 10⁻⁷), with a male predominance in both groups (71% and 64%, respectively).

Lung adenocarcinoma was the most common lung cancer subtype, accounting for all 7 cases (100%) in the PsP group and 52 cases (75%) in the non-PsP group.

When comparing both groups, 3 significant differences were noticed:

- The PsP group showed a significantly higher tumor burden on pre-treatment brain MRI, with a median of 364 mm² (IQR: 134-953) versus 110 mm² (IQR: 0-330) in the non-PsP group (*p* = 4.10 × 10⁻¹⁰).
- PDL1 expression was higher in the PsP group with a median PDL1 expression of 70% in the PsP group vs 35 % in the non PsP group (*p* < 0.001).
- The total radiation dose was significantly greater in the PsP group, with a median of 40 Gy (IQR:28-65) versus 30 Gy (IQR:27-53.25) in the non-PsP group (*p* = 3.3 × 10⁻⁷).

No significant association was found between PsP and the type of radiotherapy (*p* = 0.48), or the type of ICI administered (*p* = 0.34).

Progression-free survival (PFS), overall survival (OS), and time to next intervention (TTNI) were all numerically longer in the PsP group compared to the non-PsP group, although none of the differences reached statistical significance. Median PFS was 12.8 vs. 7.8 months (HR 0,43; 95% CI 0.15-1.19, *p* = 0,1), median OS was 17.3 vs. 14.9 months (HR 0,75; 95% CI 0.27- 2.09, *p* = 0,58), and median TTNI was 16.1 vs. 9.3 months (HR 0.48; 95% CI 0.1156-2.025, *p* = 0,32) (Supplementary Figure 2a–c).


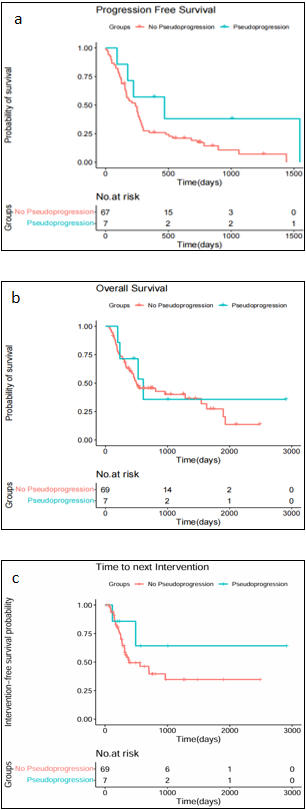


**Supplementary Fig. 2** Progression-free survival (a), overall survival (b), and time to next intervention (c) in patients with and without PsP, in the lung cancer subgroup.
